# State of the interface between conservation and physiology: a bibliometric analysis

**DOI:** 10.1093/conphys/cou003

**Published:** 2014-02-25

**Authors:** Robert Lennox, Steven J. Cooke

**Affiliations:** Fish Ecology and Conservation Physiology Laboratory, Department of Biology, Carleton University, Ottawa, Ontario, Canada K1S 5B6

**Keywords:** Bibliometrics, biodiversity conservation, conservation physiology, physiology

## Abstract

Contemporary conservation science benefits from the perspectives of a variety of different disciplines, including a recent synergy with physiology, an interface known as ‘conservation physiology’. To evaluate the degree of interaction between conservation and animal/plant physiology, we conducted three bibliometric analyses. We first pursued the use of the term ‘conservation physiology’ since its first definition in 2006 to determine how frequently it has been used and in which publications. Secondly, we evaluated the occurrence of conservation terms in animal and plant physiology journals, physiological terms in conservation journals, and a combination of terms in ecology journals. Thirdly, we explored trends in a subset of conservation physiology articles published between 2006 and 2012. We identified a surge in the use of the term ‘conservation physiology’ in 2012, after only a slow increase in usage between 2006 and 2011. Conservation journals tend to have been significantly more active in publishing conservation physiology than animal physiology, plant physiology or ecology journals. However, we found evidence that ecology and animal physiology journals began to incorporate more conservation physiology after 2006, while conservation- and plant physiology-themed journals did not. Among 299 conservation physiology articles that we identified, vertebrate taxa have been over-represented in conservation physiology compared with their relative taxonomic abundance, invertebrate taxa have been under-represented, and plants have been represented in proportion to their relative taxonomic abundance; however, those findings are reasonably consistent with publication trends in conservation biology. Diffuse distribution of conservation physiology papers throughout the literature may have been a barrier to the growth of the subdiscipline when the interface was emerging. The introduction of the focused journal *Conservation Physiology* in 2013 may address that deficiency. Moreover, development of a unifying framework could help to aggregate knowledge and attract potential contributors by highlighting and facilitating access to and application of conservation physiology.

## Abstract (precis)

A bibliometric analysis of contemporary peer-reviewed literature was used to examine the overlap between conservation and physiology. Although the term “conservation physiology” rarely appears in papers that combine the disciplines, they are indeed being integrated in recent years and we expect increased integration in the near future.

## Introduction

Conservation science was established to confront the global crisis of biodiversity loss (Soulé, 1986), but what is now a complex and widespread problem must be confronted with changing perspectives and novel approaches (Salafsky *et al.*, 2002). Thus, conservation science has become a multi-disciplinary domain with diverse subdisciplines (Soulé, 1985; Fazey *et al.*, 2005), such as conservation genetics (Frankham, 1995), conservation social science (Mascia *et al.*, 2003), conservation behaviour (Sutherland, 1998) and others. Each of these subdisciplines of conservation science provides insight to environmental management but each is most useful when combined to address particularly complex problems from different perspectives (Campbell, 2005; Balmford and Cowling, 2006). Further integrated approaches to conservation have included consideration of physiological mechanisms and have led to the development of another synergy, namely conservation physiology (Wikelski and Cooke, 2006).

The history of conservation physiology was reviewed by Cooke *et al.* (2013), who also provided an updated definition, as follows: ‘an integrative scientific discipline applying physiological concepts, tools, and knowledge to characterizing biological diversity and its ecological implications; understanding and predicting how organisms, populations, and ecosystems respond to environmental change and stressors; and solving conservation problems across the broad range of taxa (i.e. including microbes, plants, and animals)’. Several papers have articulated that conservation physiology can make important contributions to conservation science, largely by establishing cause-and-effect relationships and generating solutions therefrom (Wikelski and Cooke, 2006; Cooke and O'Connor, 2010; Seebacher and Franklin, 2012; Cooke *et al.*, 2013), but in spite of the potential of this developing subdiscipline, it is uncertain whether calls for further integration are being met by increased assimilation of physiology and conservation.

It is unclear how the development of conservation physiology has progressed in recent years and whether improved understanding of the potential for conservation physiology and a formalized definition have coincided with an increase in research consistent with the definition. The establishment of the first definition for conservation physiology occurred relatively recently (see Wikelski and Cooke, 2006) and was concurrent with a field physiology toolbox that was rapidly expanding [e.g. non-lethal biopsy (Fossi *et al.*, 1999; Cooke *et al.*, 2004), faecal hormone analyses (Schwarzenberger, 2007; Sheriff *et al.*, 2011), biotelemetry (Cooke *et al.*, 2004) and biologging (Block, 2005) and mobile respiration apparatus for plant roots, branches and foliage (Hermle *et al.*, 2010; Sayer and Tanner, 2010)] and increasing the ways and ease with which physiological data could be collected and applied to conservation problems. As such, it should follow that the incorporation of conservation physiology within scientific literature will have increased since the seminal publication by Wikelski and Cooke (2006) and other papers advocating the application of physiology to address conservation problems (e.g. Carey, 2005; Tracy *et al.*, 2006).

This study examines trends in conservation physiology publication via a bibliometric analysis of scientific literature using Thomson Scientific's Web of Science. Our question was whether conservation and physiology are becoming more frequently integrated in major journals. We attempted to address the question by conducting three searches of Thomson Scientific's Web of Science. The bibliometric analyses resulting from the three independent Web of Science searches aim to quantify whether inclusion of the term ‘conservation physiology’ is increasing within published literature through time, to determine whether conservation physiology is more frequently incorporated by major conservation, physiology or ecology journals and to identify trends in conservation physiology publications, such as typical focal taxa and type of study. In doing so, we endeavour to identify areas where conservation physiology has been active and where there is potential for increased focus. A similar examination was recently completed for conservation behaviour (i.e. Angeloni *et al.*, 2008), where the authors noted that despite interest in combining behaviour and conservation (Sutherland, 1998; Buchholz, 2007), there remained a disconnect between behaviour and conservation sciences (Caro, 2007; Caro and Sherman, 2011).

## Approach

In order to evaluate the present state of the conservation–physiology interface, we conducted a bibliometric literature survey using Thomson Scientific's Web of Science (Thomson Scientific, 2013). Web of Science provided access to journal articles and enabled us to thematize papers and identify publication trends relevant to our aims. We selected Web of Science because it provides point-in-time analysis that is easily repeatable. In addition, Web of Science allows users to collate article details in downloadable form, facilitating bibliometric analyses. We conducted our survey up to and including the year 2012 using three different searches for selected words within the ‘topic’ of the article. In the Web of Science search engine, the ‘topic’ encompasses the title, key words, key words plus (additional relevant but overlooked words to identify the article, as determined by the editors of Web of Science) and abstract of an article. An article ‘topic’ is a standard search field used in bibliometry (Angeloni *et al.*, 2008; Thomson Scientific, 2013). While searching only article ‘topics’ inherently limits searches to articles that prominently feature the search terms, it does allow for the identification of articles that are most relevant to the search terms while allowing researchers most efficiently to sort through a large number of potentially relevant articles incorporated within the Web of Science database.

Our first search of the Web of Science was the term ‘conservation physiology’. This was performed to quantify the history of usage of this term and was conducted by searching for articles that included the term in the ‘topic’ of the article. The literature survey was conducted from 1 June 2013 to 1 September 2013.

Our second search was conducted to identify integration of physiology and conservation within major conservation biology, animal physiology, plant physiology and general ecology publications. From a plethora of potentially relevant and high-impact publications in each of the four domains, we sought journals that focused on macro processes and those whose mission statement did not explicitly exclude concepts associated with conservation physiology. From this criterion, we identified 16 influential scientific publications, four representatives each of conservation, animal physiology, plant physiology and ecology within which to evaluate integration of conservation and physiology. Admittedly, this exercise of identifying target journals was somewhat subjective and was based largely on the senior author's experience rather than any quantitative measures. In conservation journals (*Biological Conservation*, *Conservation Biology*, *Global Change Biology* and *Biodiversity and Conservation*), we measured integration by searching for terms related to physiology (i.e. physiolog-, stress-, energy-, mechanis-, threshold, condition-), in animal physiology journals (*Journal of Comparative Physiology*, *Physiological and Biochemical Zoology*, *Comparative Biochemistry and Physiology* and *Journal of Experimental Biology*) and plant physiology journals (*New Phytologist*, *Journal of Experimental Botany*, *Journal of Plant Physiology* and *Plant Physiology*), we searched for terms relating to conservation (i.e. endanger-, imperil-, conserv-, restor-, manage-, poli-, threat-, decision-making), and in ecology journals (*Ecology*, *Oecologia*, *Functional Ecology* and *American Naturalist*), we searched for any combinations of the conservation and physiology terms. There were other candidate conservation (e.g. *Animal Conservation*) and ecology journals (e.g. *Journal of Animal Ecology*); however, it was necessary to select journals that included coverage of all taxa. The search terms that we employed to represent conservation and physiology were selected for their propensity to identify articles that incorporated conservation and/or physiology while minimizing false positives that did not relate to our aims.

After identifying the integration of conservation physiology among journals with different mandates, we conducted a broader search of the Web of Science for articles that could be analysed for focal taxon, publication year and document type (i.e. review paper, meta-analysis, research article). In this final analysis, we searched the Web of Science for any articles appearing in any journal within the 5 years after the first formal publication of the term ‘conservation physiology’ (i.e. between 2007 and 2012) that simultaneously related to both conservation and physiology. This search was conducted by identifying articles that included one or more conservation terms (from the following list: endanger-, imperil-, conserv-, restor-, manage-, poli-, threat-, decision-making) and also one or more physiological term (from the following list: physiolog-, stress-, energy-, mechanis-, threshold, condition) in the ‘topic’. The results of this search were refined to include only those in the Web of Science categories ‘physiology’ and ‘biodiversity and conservation’. The 3225 resulting articles were manually filtered to remove spurious hits, generating a list of 299 articles relevant to conservation physiology. The resulting list represented a subset of conservation physiology articles that we used to identify trends in conservation physiology by categorizing papers by taxon, year and document type.

## Findings

### Has the term ‘conservation physiology’ increased in prevalence in publications since 2006?

**Figure 1:**
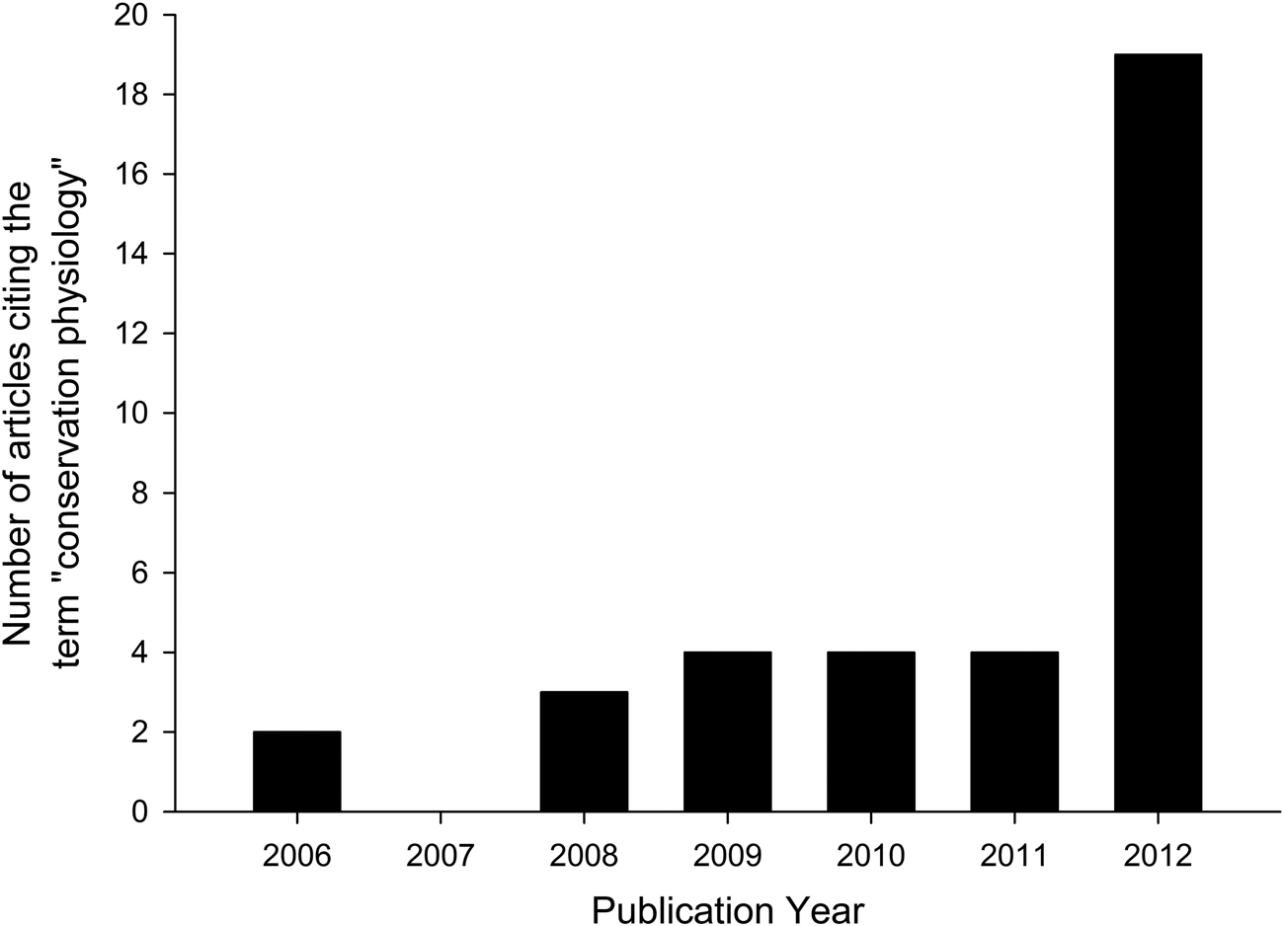
Instances of the term ‘conservation physiology’ in published scientific literature (*n* = 36).

A search for the term ‘conservation physiology’ across all years in Web of Science (Thomson Scientific, 2013) produced 36 research articles that used the term in the ‘topic’ of the article, beginning with Wikelski and Cooke (2006). Thereafter, the frequency of papers with ‘conservation physiology’ in the ‘topic’ remained relatively unchanged until 2012, in which year 19 articles were published (Fig. 1). This increase was driven largely by articles in a special issue of *Philosophical Transactions of the Royal Society B Biology* in 2012, which focused on conservation physiology and contributed nine articles to our search (although of the 12 published in the special issue, three were not captured by our search string) for that year. The 36 articles appeared in 24 different journals, with *Philosophical Transactions* contributing the most (25%; all in the special issue). No other journal exceeded two articles containing ‘conservation physiology’ in the ‘topic’.

### What is the extent of overlap between conservation and physiology in major journals?

**Figure 2:**
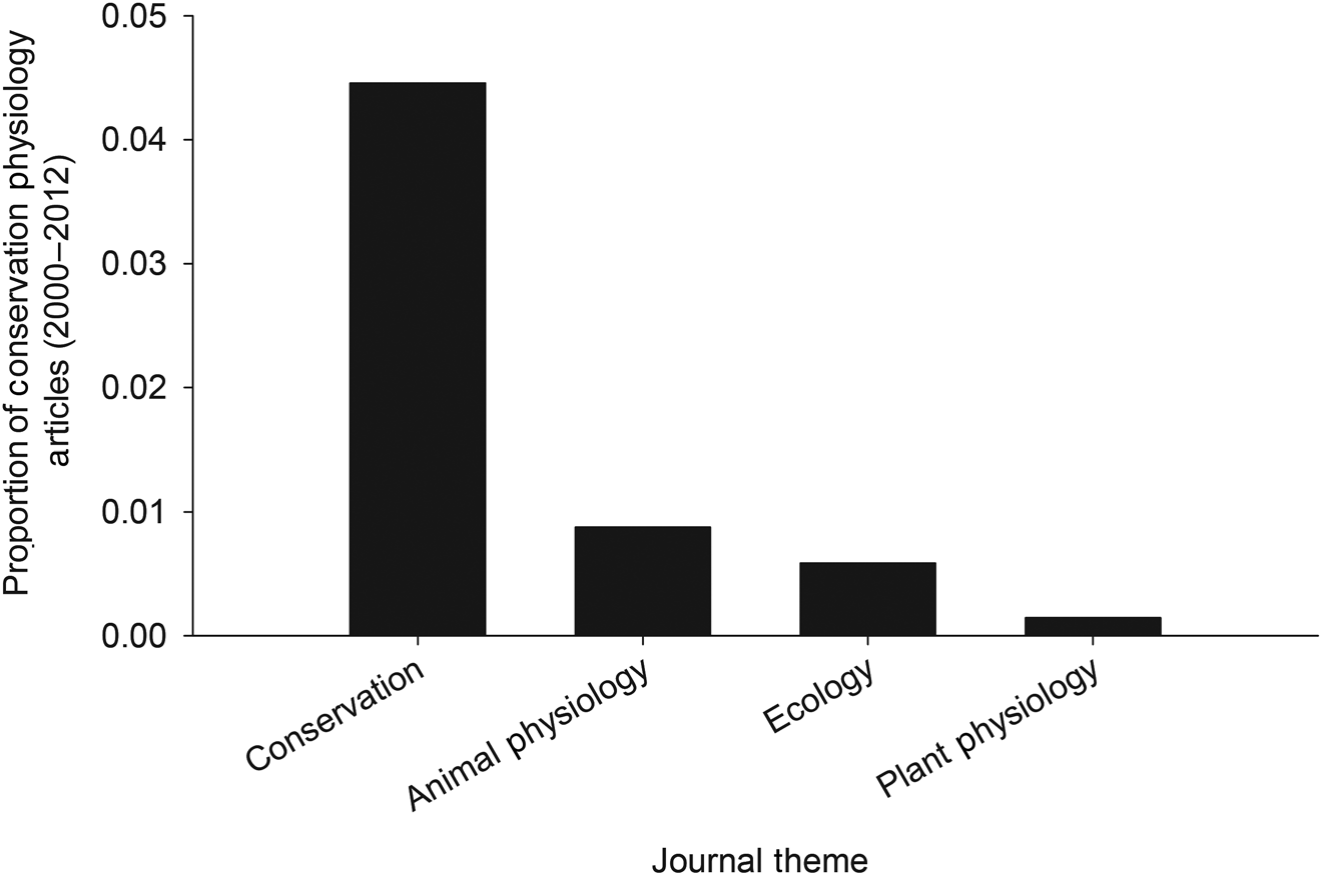
Frequency of integration between conservation and physiology in selected journals (see Fig. 3) representing animal physiology, plant physiology, ecology and biodiversity conservation. Integration was assessed by seeking conservation terms in physiology journals and vice versa, and for a combination of conservation and physiology terms in ecology journals.

**Figure 3:**
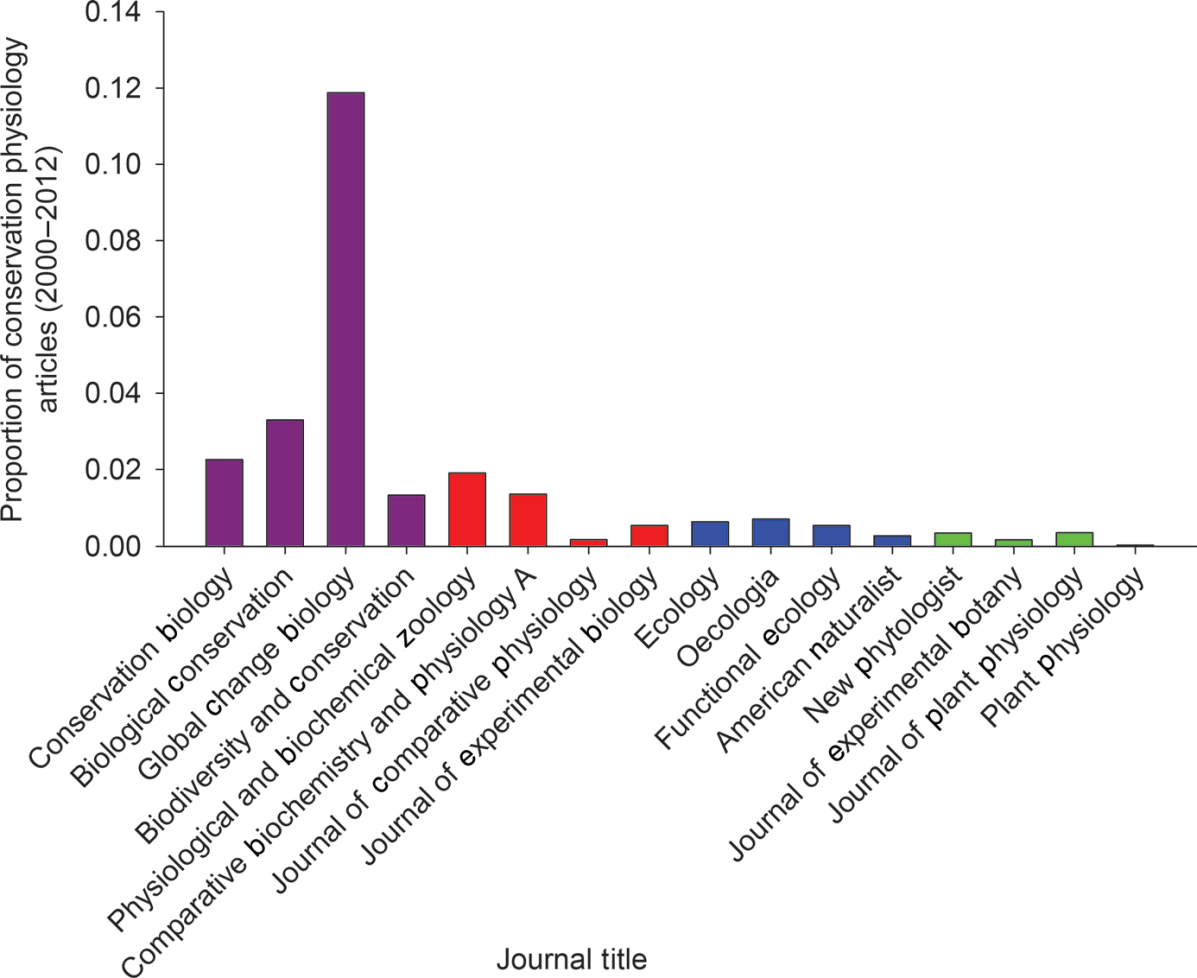
A breakdown of conservation–physiology integration in the journals identified. Four journals from each of biodiversity conservation (purple), animal physiology (red), plant physiology (green) and ecology (blue) were selected to cross-reference the integration of conservation physiology within the journal between 2000 and 2012.

**Figure 4:**
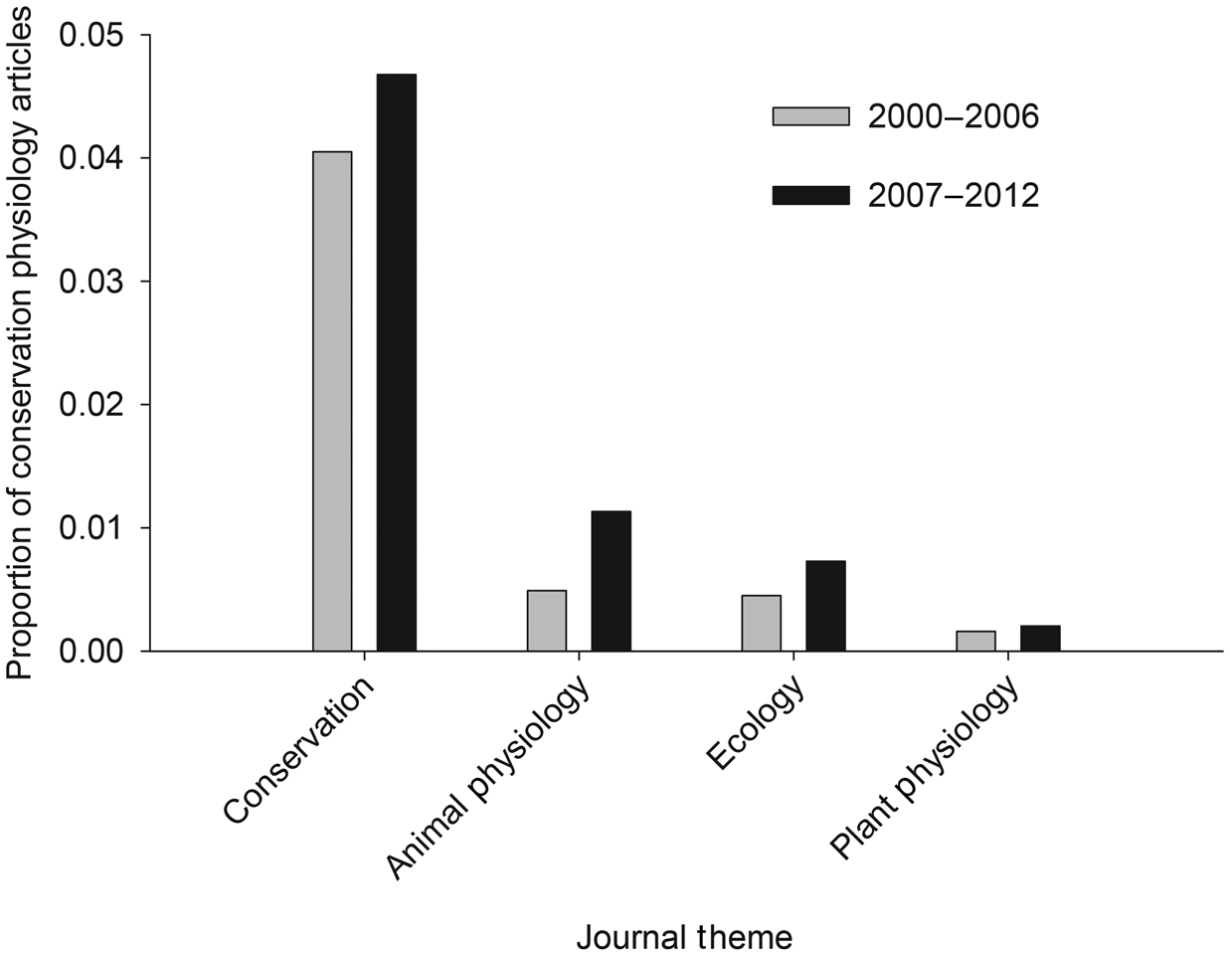
Frequency of integration between conservation and physiology in selected journals (see Fig. 3) representing animal physiology, plant physiology, ecology and biodiversity conservation in the time period 2000–2006 compared with 2007–2012. Integration was assessed by searching for conservation terms in physiology journals and vice versa, and for a combination of conservation and physiology terms in ecology journals.

Evaluation of key conservation and physiology journals revealed slow or nominal expansion of the interface between conservation and physiology. Conservation journals included a higher percentage of articles with physiological terms in the ‘topic’ (4.38% of 11 845 article) than animal physiology journals (0.80% of 12 433 articles; *z* = 12.80, *P* < 0.01; Fig. 2) or plant physiology journals (0.73% of 5903 articles; *z* = 13.08, *P* < 0.01; Fig. 2) that included conservation terms. Among the conservation journals, *Global Change Biology* was the most active at the conservation physiology interface (11.88% of 2492 articles; Fig. 3); only *Biological Conservation* also exceeded 3% overlap (3.30% of 3852 articles). *Physiological and Biochemical Zoology* had the highest percentage of overlapping articles among animal physiology-themed journals (1.91% of 1102 articles), and *New Phytologist* and *Journal of Plant Physiology* (0.35% of 4323 articles and 0.35% of 2548 articles, respectively) recorded the highest overlap among plant physiology journals (Fig. 3). Overlap between conservation and physiology increased in both conservation and physiology journals from 2000–2006 compared with 2007–2012, but the increase was significant only in animal physiology journals (plant, *z* = −0.682, *P* = 0.50; conservation biology, *z* = −1.67, *P* = 0.09; and animal physiology, *z* = 4.41, *P* < 0.01; Fig. 4).

Ecology journals published the smallest percentage of articles with conservation and physiology coinciding in the ‘topic’ (0.59% of 12 113 articles), with the journal *Ecology* publishing the highest percentage (0.64% of 4353 articles; Fig. 3). Ecological journals published a significantly smaller percentage of conservation physiology articles relative to both conservation journals (*z* = −18.635, *P* < 0.01) and animal physiology journals (*z* = 2.175, *P* = 0.03), but more than the plant physiology journals (*z* = 5.939, *P* < 0.01; Fig. 2). The observed increase in the proportion of conservation physiology after 2006 was significant (*z* = −2.02, *P* = 0.04; Fig. 4).

### What trends exist in the conservation physiology literature?

**Figure 5:**
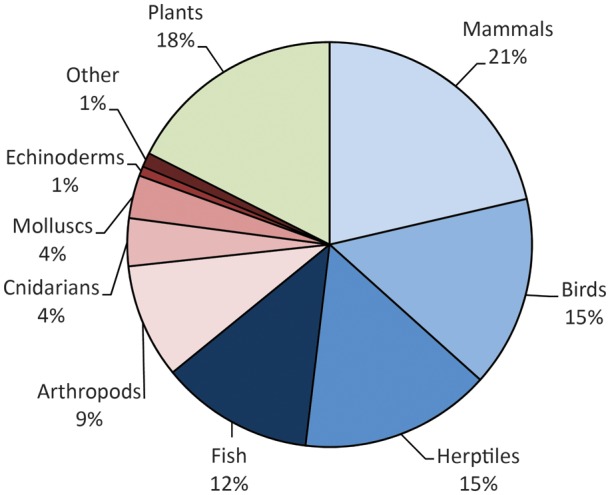
Taxonomic representation in the conservation physiology literature. A search for publications addressing conservation physiology produced a subset of conservation physiology publications between 2007 and 2012; 262 of the 299 resulting papers addressed a specific taxonomic group. Focal taxa are described in the pie chart; blue hues represent vertebrate taxa (64%) while red hues represent invertebrate taxa (18%) and green represents plants (18%).

A subset of conservation physiology articles retrieved from Web of Science included 299 articles that were identified by including both conservation- and physiology-oriented key words in the ‘topic’. These 299 articles came from 42 different scientific publications, most frequently *Biological Conservation* (15%), *Global Change Biology* (13%) and *Animal Conservation* (7%), with *Physiological and Biochemical Zoology* contributing the most among physiology-themed journals (4%). Thirty-two of the 42 journals contributed multiple conservation physiology articles to the database. Most of the publications identified were considered research articles (91%), with some reviews (7%) and three meta-analyses (1%). Among papers that were investigating individual species or taxa, vertebrates were the most heavily represented taxon (64%), within which mammals (33%), herpetofauna (24%), birds (23%) and fishes (19%) were represented to a similar extent. Plants and invertebrates each made up 18% of the articles. Invertebrate articles focused primarily on insects or other arthropods (50%), corals or other cnidarians (21%) and molluscs (19%; Fig. 5). ‘Climate change’ and/or ‘global warming’ were ‘topics’ within 20% of the papers, while ‘stress’ or ‘-cort-’ (e.g. glucocorticoids) were in the title, key words or abstract of 49% of the 299 publications. The year 2011 contained the highest percentage of all published articles (23%), followed by 2012 (21%), representing a steady increase from earlier years (2007, 10%; 2008, 12%; 2009, 14%; and 2010, 18%). Only 2% of the articles referred to ‘conservation physiology’ within the ‘topic’.

## Synthesis

We acknowledge some of the limitations of our methods used to explore trends in the interface between conservation and physiology. We did not include all search terms that could possibly relate to conservation or to physiology, we selected only four representative journals for each of conservation, ecology, plant physiology and animal physiology and we were limited to searching for terms in the ‘topic’ of articles. However, there have previously been no attempts to evaluate the extent of integration between conservation and physiology since Wikelski and Cooke (2006) coined the term and provided a foundation for the discipline, and our results elucidate some interesting trends within conservation physiology published to date. We expected that greater integration of conservation and physiology would manifest in the literature review as increased focus on conservation in animal and plant physiology journals, increased focus on physiology in conservation journals and increased coincidence of physiology and conservation terms in ecology journals over time. However, this was largely not the case. Based on our literature survey, expansion of conservation physiology in prominent conservation, physiology and ecology journals to date appears to have been relatively slow, despite much opportunity (Cooke *et al.*, 2013).

Major conservation journals were more active at the conservation–physiology interface than the major physiology and ecology journals we analysed. *Global Change Biology* (a conservation journal) was especially active; articles therein frequently addressed the effects of human disturbance on flora, including mentions of energetic, thermal or water stress as a result of ecosystem degradation or pollution. Conversely, physiology journals were relatively unlikely to incorporate articles that addressed conservation issues. Caro and Sherman (2013) surveyed ethologists to understand their reluctance to contribute to conservation, concluding that many felt they were not specialized enough to make conservation statements, were disinterested in or disenchanted by conservation, or felt that their contributions would be unrecognized or were beyond the scope of their research interest. If similar attitudes toward conservation exist among physiologists, it would explain the lack of integration that we identified in physiology journals. Such barriers can be breached effectively by collaboration between scientists with different specialties and by improved understanding of conservation physiology and its role within conservation science, of which there are many advantages, including new avenues for funding for physiologists that make their research applicable to conservation science. Many conservation entities may be reluctant to authorize physiological sampling protocols that are perceived as invasive, making it difficult to apply physiology even if it could provide information that would be important for conservation. However, there are a growing number of non-invasive sampling strategies (e.g. faecal glucocorticoids) that do not require the handling of wildlife.

Our finding that conservation physiology has only recently (i.e. 2012) experienced significant expansion following the publication by Wikelski and Cooke (2006) contradicts the vast potential for evidence-based management afforded by insight into animal physiology. In an animal behaviour review by Sutherland (1998), it was conjectured that 10% of animal behaviour articles should be applicable to conservation, although Angeloni *et al.* (2008) found integration to be <0.5%. While 10% is an optimistic target for integration of conservation with other sciences, we found the rate of integration between conservation and physiology to be closer to 2% within the journals we identified. We offer the following two possible explanations for this observation: either the conservation–physiology interface is relatively saturated and, contrary to our expectations, had limited growth potential prior to Wikelski and Cooke (2006) or, alternatively, conservation physiology is having difficulty becoming established as an autonomous subdiscipline and is not attracting potential contributors. However, we reject the premise of the first explanation, because advancements in the conservation physiology toolbox have opened up new avenues for physiological research and, as such, there should indeed be ample growth potential that has yet to be realized within conservation physiology.

According to our results, integration between conservation and physiology is increasing and, indeed, further integration could manifest in the near future. Following the special issue of *Philosophical Transactions of the Royal Society B Biology* in 2012, an additional special issue dealing with conservation physiology is ‘in press’ with *Physiological and Biochemical Zoology* as well as the inaugural issue of a focused online journal, *Conservation Physiology*, published in 2013. The special issues and the focused journal aggregate research being conducted relating to conservation physiology, improving accessibility to conservation physiology papers and drawing attention to the subdiscipline.

Our search string in the Web of Science identified a number of articles that have considered the conservation applications afforded by physiological research in a large number of journals representing a variety of disciplines, such as physiology, conservation and ecology, as well as those that were taxon specific, which have previously incorporated conservation physiology. This exposed a wide audience with different research interests to the principles of conservation physiology; however, this audience would infrequently encounter instances of conservation physiology unless they were scanning many different publications. Special issues have been delivered and have improved the exposure of the scientific community to conservation physiology, and nine of the 12 articles in the 2012 special issue of *Philosophical Transactions of the Royal Society B Biology* were review articles aggregating existing conservation physiology studies.

Moving forward, a focused journal should improve accessibility to research in conservation physiology, attracting researchers who have conducted studies with integrated applications, and implicitly identifying opportunities for future directions in conservation physiology for researchers within a single outlet. Indeed, many researchers are probably unaware of whether they are conducting research that falls under the auspice of ‘conservation physiology’. For example, three of the 12 journals in the special issue of *Philosophical Transactions of the Royal Society B Biology* were not among the 3225 conservation physiology-themed articles identified in the Web of Science search, which was conducted to identify instances of conservation physiology between 2007 and 2012. This is a limitation of the ‘topic’ search function in Web of Science, because articles that address physiology in the body of the article but not in the ‘topic’ would not be identified by the search.

Integrations with conservation science have a conceptual framework upon which the integration is based, such as conservation behaviour (i.e. Tinbergen's four questions; Buchholz, 2007) and, indeed, a framework specific to the integration of behaviour and conservation was recently published (Berger-Tal *et al.*, 2011). In the near future, an integrated framework for conservation physiology should be developed to streamline and direct research that may enter the new subdiscipline (Cooke *et al.*, 2014). Although the ‘physiology/life-history nexus’ conceived by Ricklefs and Wikelski (2002) has provided direction for many early studies in conservation physiology (Cooke *et al.*, 2014), the breadth of the subdiscipline merits a distinct unifying framework. Indeed, Berger-Tal *et al.* (2011) state that the lack of a unifying framework can make a discipline appear disorganized and inaccessible to potential contributors. A lack of unifying framework could explain why we had difficulty in identifying articles that applied to conservation physiology, because articles did not always cite the term and were published in a variety of different journals in disparate research categories. We argue that the addition of a unifying framework to conservation physiology should be a next step for the development of the interface.

As conservation physiology begins to become entrenched as an important branch of conservation science, future researchers should take note of some discrepancies existing within the field. For example, we found there to be some focus on certain taxa among conservation physiology papers and disproportionate attention towards vertebrates, which is in fact a common trend within the larger body of conservation science; in *Conservation Biology* and *Biological Conservation*, Clark and May (2002) found that vertebrates are heavily over-represented by 69% of the conservation literature while invertebrates are poorly represented. Thus, the biases towards some taxa in our conservation physiology literature survey were consistent with overall trends in conservation biology. Some of the bias may reflect the reality that physiological knowledge and tools developed for humans are often transferrable to other vertebrates, but not necessarily to other taxa. In addition, there is a bias in conservation biology where efforts focus on sympathetic fauna for which access to funding can be relatively high, those species that are economically important or culturally valuable, or simply those species for which public awareness and compassion are relatively high.

Conservation physiology existed well before the term was first coined (Wikelski and Cooke, 2006), with integration occurring since at least Rachel Carson's influential *Silent Spring* (Carson, 1962), in which the toxic effects of DDT were related to declining raptor abundance. The recent formal classification of a subdiscipline was intended to increase the frequency with which such links between organism physiologies are related to conservation and management. Although there was a significant increase in conservation-themed articles in animal physiology journals after 2006 compared with the period 2000–2006, physiology-themed journals have tended to be relatively unlikely to publish articles using words relevant to conservation, demonstrating a potential lag among physiologists in participating in conservation research. This could be attributable to difficulty in accessing animals of conservation concern for physiological assessment, difficulty bridging the gap between conservation and physiology, or perhaps a disinterest or disenchantment with conservation among physiologists as in Caro and Sherman (2013). Conservation physiology is still ‘in development’, and a major hurdle to further assimilation of conservation and physiology is increased contribution from physiologists.

Conservation physiology has the potential to provide the foundation for evidence-based conservation and management. Recent efforts to consolidate the field of conservation physiology should help to aggregate research that has been spread among many journals in disparate fields, which are not always exposed to researchers outside of their field. A focused journal and recent special issues may promote integration between the two domains, but it will be more important that conservation physiologists improve accessibility to their research for environmental managers. In fact, conservation physiologists should consider environmental managers to be a primary target audience when designing studies and discussing results, thereby improving the likelihood that their research has management relevance. Focusing on success stories (see Cooke *et al.*, 2012) while also being transparent regarding the weaknesses and limitations of physiology (see Cooke and O'Connor, 2010) will be essential to increasing integration between conservation and physiology going forward.

## References

[1] Angeloni L Schlaepfer MA Lawler JJ Crooks KR (2008) A reassessment of the interface between conservation and behaviour. Anim Behav 75: 731–738.

[2] Balmford A Cowling RM (2006) Fusion or failure? The future of conservation biology. Conserv Biol 20: 692–695. 16909557 10.1111/j.1523-1739.2006.00434.x

[3] Berger-Tal O Polak T Oron A Lubin Y Kotler BP Saltz D (2011) Integrating animal behavior and conservation biology: a conceptual framework. Behav Ecol 22: 236–239.

[4] Block BA (2005) Physiological ecology in the 21st century: advancements in biologging science. Integr Comp Biol 45: 305–320. 21676774 10.1093/icb/45.2.305

[5] Buchholz R (2007) Behavioural biology: an effective and relevant conservation tool. Trends Ecol Evol 22: 401–407. 17590477 10.1016/j.tree.2007.06.002

[6] Campbell LM (2005) Overcoming obstacles to interdisciplinary research. Conserv Biol 19: 574–577.

[7] Carey C (2005) How physiological methods and concepts can be useful in conservation biology. Integr Comp Biol 45: 4–11. 21676738 10.1093/icb/45.1.4

[8] Caro T (2007) Behavior and conservation: a bridge too far? Trends Ecol Evol 22: 394–400. 17590476 10.1016/j.tree.2007.06.003

[9] Caro T Sherman PW (2011) Endangered species and a threatened discipline: behavioural ecology. Trends Ecol Evol 26: 111–118. 21257224 10.1016/j.tree.2010.12.008

[10] Caro T Sherman PW (2013) Eighteen reasons animal behaviourists avoid involvement in conservation. Anim Behav 85: 305–312.

[11] Carson R (1962) Silent Spring. Houghton Mifflin Harcourt, Boston.

[12] Clark JA May R (2002) Taxonomic bias in conservation research. Science 297: 191–192. 12117005 10.1126/science.297.5579.191b

[13] Cooke SJ O'Connor CM (2010) Making conservation physiology relevant to policy makers and conservation practitioners. Conserv Lett 3:159–166.

[14] Cooke SJ Hinch SG Wikelski M Andrews RD Wolcott TG Butler PJ (2004) Biotelemetry: a mechanistic approach to ecology. Trends Ecol Evol 19: 334–343. 16701280 10.1016/j.tree.2004.04.003

[15] Cooke SJ Hinch SG Donaldson MR Clark TD Eliason EJ Crossin GT Raby GD Jeffries KM Lapointe M Miller K, et al. (2012) Conservation physiology in practice: how physiological knowledge has improved our ability to sustainably manage Pacific salmon during up-river migration. *Philos Trans R Soc Lond B Biol Sci* 367: 1757–1769. 10.1098/rstb.2012.0022 PMC3350662 22566681

[16] Cooke SJ Sack L Franklin CE Farrell AP Beardall J Wikelski M Chown SL (2013) What is conservation physiology? Perspectives on an increasingly integrated and essential science. Conserv Physiol 1: doi:10.1093/conphys/cot001. 10.1093/conphys/cot001 PMC4732437 27293585

[17] Cooke SJ Blumstein DT Buchholz R Caro T Fernández-Juricic E Franklin CE Metcalfe J O'Connor CM St Clair CC Sutherland WJ (2014) Physiology, behaviour and conservation. Physiol Biochem Zool 87: 1–14. 24457917 10.1086/671165

[18] Fazey I Fischer J Lindenmayer DB (2005) What do conservation biologists publish? Biol Conserv 124: 63–73.

[19] Fossi MC Casini S Marsili L (1999) Nondestructive biomarkers of exposure to endocrine disrupting chemicals in endangered species of wildlife. Chemosphere 39: 1273–1285. 10467722 10.1016/s0045-6535(99)00195-2

[20] Frankham R (1995) Conservation genetics. Annu Rev Genet 29: 305–327. 8825477 10.1146/annurev.ge.29.120195.001513

[21] Hermle S Lavigne MB Bernier PY Bergeron O Paré D (2010) Component respiration, ecosystem respiration, and net primary production of a mature black spruce forest in northern Quebec. Tree Physiol 30: 527–540. 20215120 10.1093/treephys/tpq002

[22] Mascia MB Brosius JP Dobson TA Forbes BC Horowitz L McKean MA Turner NJ (2003) Conservation and the social sciences. Conserv Biol 17: 649–650.

[23] Ricklefs RE Wikelski M (2002) The physiology/life-history nexus. Trends Ecol Evol 17: 462–468.

[24] Salafsky N Margoluis R Redford KH Robinson JG (2002) Improving the practice of conservation: a conceptual framework and research agenda for conservation science. Conserv Biol 16: 1469–1479.

[25] Sayer EJ Tanner EV (2010) A new approach to trenching experiments for measuring root–rhizosphere respiration in a lowland tropical forest. Soil Biol Biochem 42: 347–352.

[26] Schwarzenberger F (2007) The many uses of non–invasive faecal steroid monitoring in zoo and wildlife species. Int Zoo Yearb 41: 52–74.

[27] Seebacher F Franklin CE (2012) Determining environmental causes of biological effects: the need for a mechanistic physiological dimension in conservation biology. Philos Trans R Soc Lond B Biol Sci 367: 1607–1614. 22566670 10.1098/rstb.2012.0036 PMC3350663

[28] Sheriff MJ Dantzer B Delehanty B Palme R Boonstra R (2011) Measuring stress in wildlife: techniques for quantifying glucocorticoids. Oecologia 16: 869–887. 21344254 10.1007/s00442-011-1943-y

[29] Soulé ME (1985) What is conservation biology? BioScience 35: 727–734.

[30] Soulé ME (1986) Conservation Biology. The Science of Scarcity and Diversity. Sinauer Associates Inc, Sunderland.

[31] Sutherland WJ (1998) The importance of behavioural studies in conservation biology. Anim Behav 56: 801–809. 9790690 10.1006/anbe.1998.0896

[32] Thomson Scientiﬁc (2013) Institute for Scientiﬁc Information Web of Science. Thomson Scientiﬁc, Stamford. http://isiknowledge.com (accessed June–September 2013).

[33] Tracy CR Nussear KE Esque TC Dean-Bradley K Tracy CR DeFalco LA Castle KT Zimmerman LC Espinoza RE Barber AM (2006) The importance of physiological ecology in conservation biology. Integr Comp Biol 46: 1191–1205. 21672817 10.1093/icb/icl054

[34] Wikelski M Cooke SJ (2006) Conservation physiology. Trends Ecol Evol 21: 38–46. 16701468 10.1016/j.tree.2005.10.018

